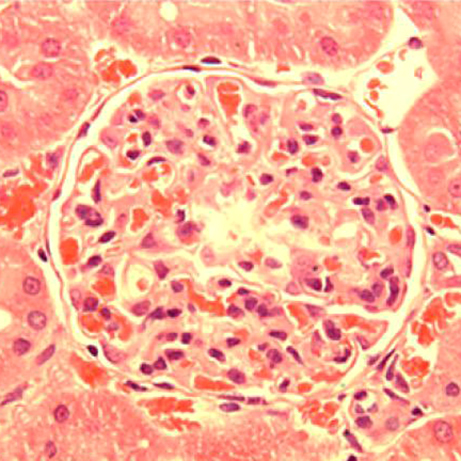# Insights into kidney disease using a bicongenic rat model

**Published:** 2013-11

Crescentic glomerulonephritis (CRGN) is a rapidly progressing kidney disease characterised by crescent-shaped scars on the glomeruli. The disease can cause potentially fatal renal failure. Experimental rat strains have been used to study CRGN at the molecular level, and this has allowed two key susceptibility loci to be mapped. One of the commonly used strains is susceptible to induction of CRGN, whereas another is resistant, allowing the effects of the susceptibility loci in different genetic backgrounds to be determined. Here, Timothy Aitman’s group establish a new bicongenic rat model in which both susceptibility loci are introgressed into the resistant strain. Interestingly, the resulting rats display macrophage-dependent CRGN in response to injection by nephrotoxin; however, they remain resistant to glomerulonephritis induced by serological autoimmunity. This work thereby provides a novel model for the study of macrophage-dependent CRGN, and also paves the way for elucidation of the mechanisms underlying susceptibility to different forms of the disease. **Page 1477**

**Figure:**